# GPs’ involvement in diagnosing, treating, and referring patients with suspected or confirmed primary cutaneous melanoma: a qualitative study

**DOI:** 10.3399/bjgpopen20X101028

**Published:** 2020-04-16

**Authors:** Andrea L Smith, Caroline G Watts, Samuel Robinson, Helen Schmid, Chiao-Han Chang, John F Thompson, Frances Rapport, Anne E Cust, Australian Melanoma Centre of Research Excellence Study Group

**Affiliations:** 1 Research Fellow, Australian Institute of Health Innovation, Macquarie University, Sydney, Australia; 2 Melanoma Institute Australia, Sydney, Australia; 3 Research Fellow, Cancer Epidemiology and Prevention Research, Sydney School of Public Health, The University of Sydney, Sydney, Australia; 4 Surveillance, Epidemiology and Prevention Program, Kirby Institute, UNSW, Sydney, Australia; 5 Research Officer, Cancer Epidemiology and Prevention Research, Sydney School of Public Health, The University of Sydney, Sydney, Australia; 6 Project Manager, Centre for Cancer Research, Westmead Institute for Medical Research, The University of Sydney, Westmead, Australia; 7 Research Assistant, Cancer Epidemiology and Prevention Research, Sydney School of Public Health, The University of Sydney, Sydney, Australia; 8 Melanoma Institute Australia, Sydney, Australia; 9 Emeritus Professor of Melanoma and Surgical Oncology, The University of Sydney, Sydney, Australia; 10 Professor of Health Implementation Science, Australian Institute of Health Innovation, Macquarie University, Sydney, Australia; 11 Professor of Cancer Epidemiology, Cancer Epidemiology and Prevention Research, Sydney School of Public Health, The University of Sydney, Sydney, Australia; 12 Melanoma Institute Australia, Sydney, Australia

**Keywords:** general practice, melanoma, qualitative research, diagnosis, treatment, Australia, primary healthcare

## Abstract

**Background:**

In Australia, melanoma is managed in primary and secondary care settings. An individual concerned about a suspicious lesion typically presents first to their GP.

**Aim:**

To identify factors influencing GPs’ decisions to diagnose, treat, or refer patients with suspected melanoma.

**Design & setting:**

Semi-structured interviews were undertaken with 23 GPs working in general practice or skin cancer clinics in Australia.

**Method:**

The semi-structured interviews were audio-recorded, de-identified, and professionally transcribed. Thematic analysis was used to analyse the data.

**Results:**

Considerable variation existed in GPs’ self-reported confidence and involvement in melanoma management. Multiple factors were identified as influencing GPs’ decisions to diagnose, treat, or refer patients with suspected or confirmed melanoma. Health system level factors included the overlapping roles of GPs and specialists, and access to and/or availability of specialists. Practice level factors included opportunities for formal and informal training, and having a GP with a special interest in skin cancer within their practice. GP and patient level factors included the GP’s clinical interests, the clinical features (for example, site and size) and histopathology of the suspected melanoma, eligibility for possible sentinel lymph node biopsy, and patient preferences. For some GPs, concerns over misdiagnosis and the option of referring patients at any stage in the melanoma management continuum appeared to affect their interest and confidence in melanoma management.

**Conclusion:**

GP involvement in melanoma patient care can extend well beyond cancer screening, prevention and supportive care roles to include provision of definitive melanoma patient management. GPs with an interest in being involved in melanoma management should be encouraged and supported to develop the skills needed to manage these patients, and to refer when appropriate.

## How this fits in

In Australia, melanoma can potentially be diagnosed and treated wholly within the primary care setting, yet little is known about how GPs decide how involved they wish to be in melanoma management. This qualitative interview study reveals the diversity of GP engagement in melanoma management and highlights how GPs’ involvement can extend well beyond prevention, early detection, and supportive care to include provision of definitive melanoma management. The study identifies multiple factors influencing a GP’s decision to treat or refer a patient with melanoma. The findings indicate that more attention should be given to GPs’ involvement in melanoma management, in particular to how GPs can be further supported to deliver optimal melanoma care.

## Introduction

Melanoma care in Australia is noteworthy for several reasons. Unlike the majority of cancers, primary cutaneous melanoma can potentially be diagnosed and treated wholly within the primary care setting. This raises a unique set of challenges for Australian GPs, whose involvement in the management of other common cancers, such as lung, prostate, breast, or bowel cancer, focuses on prevention, early detection, survivorship, and end-of-life care, with definitive diagnosis and treatment being the responsibility of specialist clinicians.^1–3^

Australian GPs’ involvement in the diagnosis and treatment of melanoma may differ from melanoma management pathways in other countries. In the UK, for example, current guidelines explicitly discourage^4–6^ GP involvement in the management of patients with melanoma other than the making of an initial visual diagnosis.^7^ According to the current UK guidelines, all patients who present with a lesion that the GP suspects to be a melanoma should be referred to secondary care for diagnosis and treatment.^4–6^ In addition, current European guidelines do not make any recommendations about which health professionals should biopsy suspicious skin lesions.^8,9^ However, given the increasing incidence of melanoma in many countries around the world,^10,11^ including the UK, and the lack of specialists to meet this increasing demand, some have argued there is a benefit in learning from Australia’s experience in melanoma management, in particular GPs’ involvement in melanoma diagnosis and care.^7^

Australia has the highest incidence of melanoma in the world^10^ and it is the most frequently occurring cancer among younger Australian adults.^12^ Public health campaigns and professional education initiatives have alerted the Australian public and GPs to the importance of melanoma prevention and early detection. In New South Wales (NSW), GPs working either in mainstream general practice or in dedicated skin cancer clinics diagnose more than half of all melanomas.^13^ In Queensland, 85% of patients with melanoma see a GP at least once during the diagnostic process, usually for the initial consultation before referral to a specialist for definitive treatment.^14^ However, little is known about how Australian GPs decide two critical issues: first, which suspicious melanocytic skin lesions will they biopsy themselves and which patients they will refer to a specialist for diagnosis. Second, if they do choose to biopsy, which confirmed melanomas will they then manage definitively, namely by appropriate wide local excision, discussion of staging by sentinel lymph node biopsy if appropriate, and follow-up. What is known is that considerable variation exists in GP practice in relation to the decision to biopsy versus the decision to refer, with up to 20% of GPs reporting that they do not perform biopsies at all.^15^ However, to the authors' knowledge, there are currently no available data reporting the factors that influence a GP’s decision to manage and treat patients with melanoma themselves rather than to refer them to a specialist or to a specialist centre.

Previous research has explored various aspects of melanoma care in the Australian setting,^14,16–22^ including compliance with clinical practice guidelines,^15,23–27^ but not how GPs understand their multiple potential roles within melanoma patient care. The aim of the current study was to identify factors that affect GPs’ confidence in, and attitudes towards, managing patients with suspected or confirmed melanoma, and how GPs explain their decisions to biopsy, treat, or refer.

## Method

### Sampling and recruitment

GPs were recruited at a national GP conference in Brisbane, Queensland (October 2018) and at a skin cancer training workshop in Sydney, NSW (December 2018). GPs were eligible to take part in the study if they had worked in general practice in Australia in the previous year. A melanoma management questionnaire (reported separately) was completed by 231 GPs, of whom 104 (45%) indicated interest in being interviewed. Twenty-three GPs (22%) provided informed consent to be interviewed; they were each reimbursed $100 (AUD) for their time. Recruitment ceased when no new information was reported relating to factors influencing GPs’ decisions to diagnose, treat, or refer patients with suspected melanoma; this was also the point at which the authors were able to fully explain the themes and how they related to one another.

### Data collection and analysis

A semi-structured interview guide (Supplementary File 1) was developed following a review of the literature and discussion with a multidisciplinary team comprising clinicians (dermatologists, medical oncologists, surgeons, radiologists, GPs, and pathologists) and researchers. Telephone interviews were conducted by an experienced qualitative research officer and an experienced qualitative post-doctoral research fellow, and lasted 16–39 minutes (mean 24 minutes). They were audio-recorded, de-identified, and professionally transcribed. Transcripts were read by two members of the research team. Data were compared within and across interviews to identify commonalities, differences, and patterns. NVivo (version 12) was used to support data management and analysis. Analysis followed the principles of Braun and Clarke’s reflexive version of thematic analysis.^28,29^ Coding and theme development were conducted independently. A thematic map was developed to show how the themes related to one another. The themes and thematic map were discussed and refined with the wider research team. Reporting of the findings was informed by the consolidated criteria for reporting qualitative research (COREQ) guidelines.^30^

## Results

The sample comprised 23 GPs (12 male; 11 female) working in general practice and skin cancer clinics (independent practice *n* = 10; group medical practice *n* = 8; skin cancer clinic *n* = 4; other *n* = 1 [a general practice clinic at an Australian Defence Force site]), as shown in Table 1.

**Table 1.** Characteristics of GPs and their practice

**Characteristic**	**Frequency, *n* (%) (*n* = 23**)
Sex	
Male	12 (52)
Female	11(48)
Age group	
<30 years	2 (9)
30–49	12 (52)
50–69	9 (39)
≥70	0 (0)
Practice type	
Independent practice	10 (43)
Group medical practice	8 (35)
Skin cancer clinic	4 (17)
Other^a^	1 (4)
Number of years practising as a GP	
<5	9 (39)
6–10	5 (22)
11–20	2 (9)
>20	7 (30)
Number of patients diagnosed with invasive melanoma in the previous year	
0 patients	3 (13)
1 patient	5 (22)
2–5 patients	10 (43)
6–10 patients	5 (22)
>10 patients	0 (0)
Practice by ARIA index^b^	
Highly accessible	14 (61)
Accessible	7 (30)
Less accessible	2 (9)
Practice by SEIFA index^c^	
Q1 (most disadvantaged)	4 (17)
Q2	5 (22)
Q3	7 (30)
Q4 (least disadvantaged)	7 (30%)

a Other: a general practice clinic at an Australian Defence Force site.

b ARIA: Accessibility/Remoteness Index of Australia.

c SEIFA: Socio-Economic Indexes for Areas.

### Variation in confidence and engagement in melanoma patient care

The GPs typically viewed their primary role to be the diagnosis of melanomas. Almost all were comfortable performing skin checks and biopsies before referring to a specialist for wide local excision (three of the 23 participants reported never performing biopsies):

*‘General practitioners are the primary diagnosticians of melanomas ... we are really on the front line, examining patients, finding these moles and if need be* [referring these patients].’ (ID208;<5 years’ experience, skin cancer clinic)

**Figure 1.**
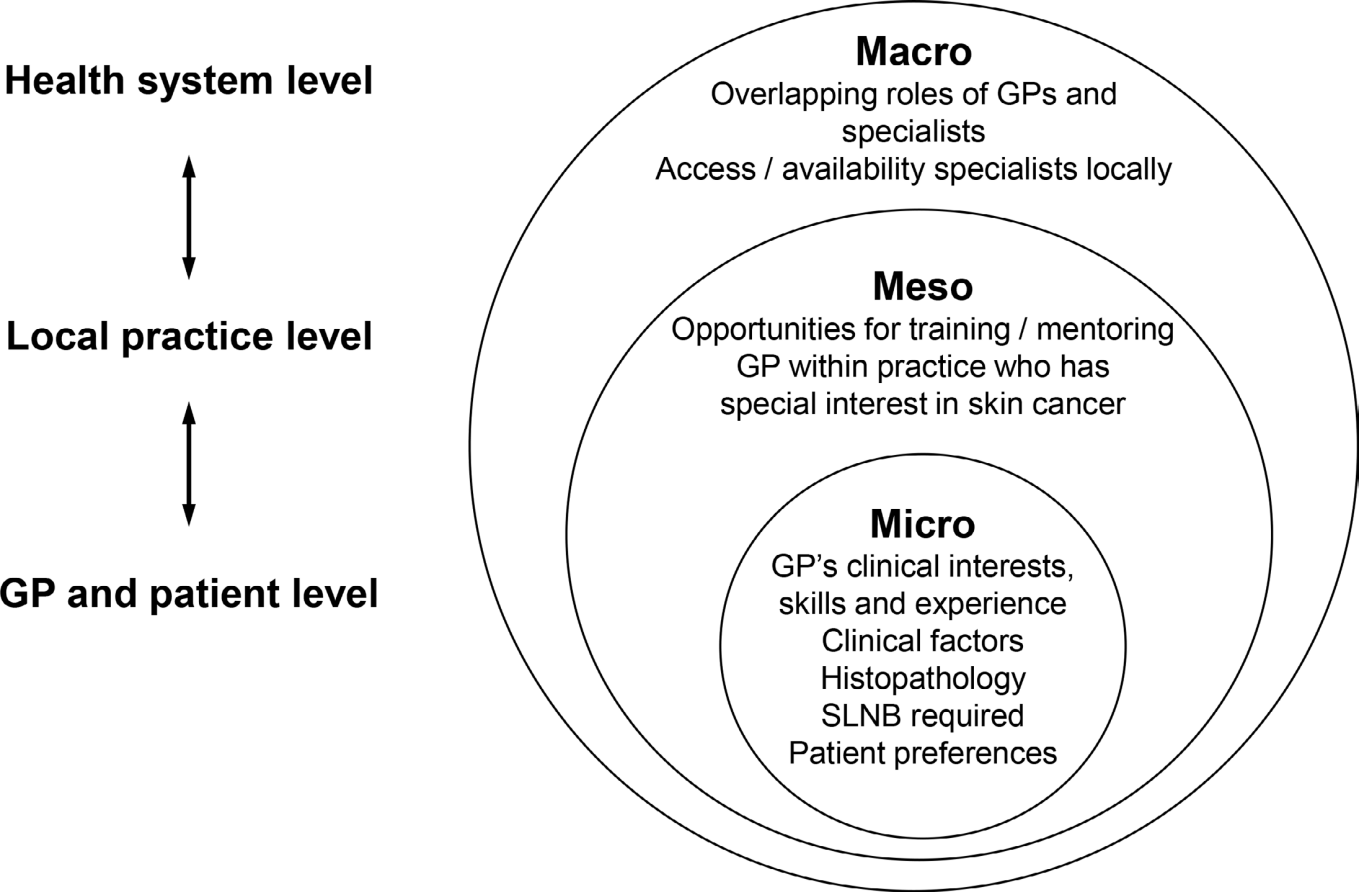
Factors affecting GPs’ confidence and engagement in melanoma management.SLNB = sentinel lymph node biopsy.

However, confidence and engagement in melanoma care varied widely. At one extreme were GPs with almost no interest or active involvement in any aspect of melanoma management; at the other, GPs who were confidently performing skin checks, taking biopsies of suspicious lesions, and performing wide local excisions. Reasons for this variation were multi-faceted and occurred at the health system (macro), practice (meso), and GP and patient (micro) levels (Figure 1; see also Table S1, for illustrative quotes to support Figure 1). The variations were not simply related to practice setting, as several GPs working in mainstream general practice appeared to be as confident at managing patients across the continuum of melanoma care as GPs working in skin cancer clinics.

The following sections examine these health system, practice, and GP and patient level factors and their influence on melanoma management and referral in more detail.

### Factors impacting on GPs’ decisions around when to refer and to whom they would refer

#### Health system (macro) level

**Figure 2.**
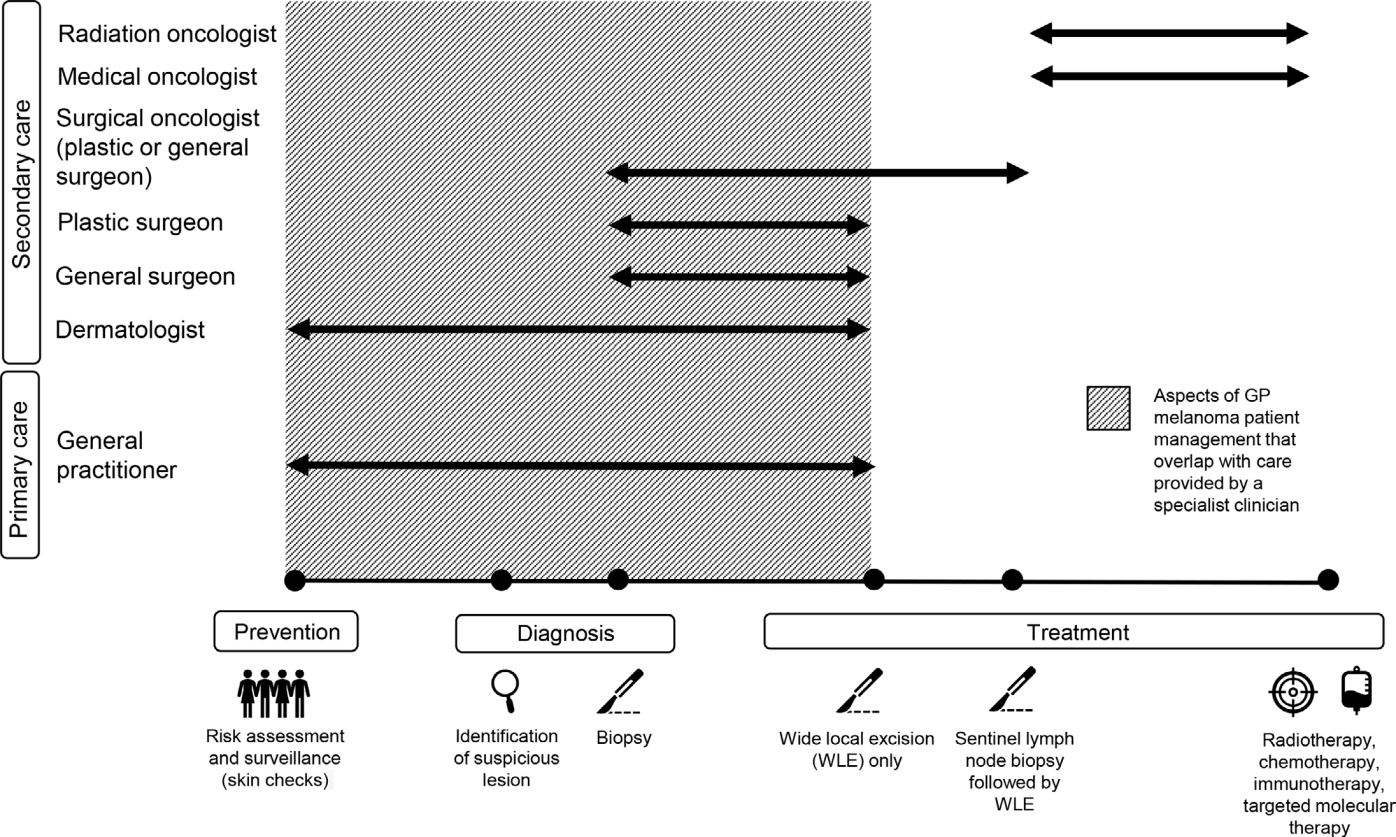
GP and specialist roles in melanoma management in Australia

At the health system level, the overlapping roles that GPs and specialists play in melanoma management meant that, for most of the GPs interviewed, a diverse range of referral options was available at multiple points along the melanoma care continuum (Figure 2).

*‘Different doctors have different levels of expertise, so while my repertoire will be initial detection and biopsy and then sorting out the referrals and supporting them, if* [patients] *need definitive surgical management, they might need a plastic surgeon or someone like that, or in terms of recognising dysplastic lesions that are just changing into melanomas that are a little bit more tricky for me to diagnose* [then] *a dermatologist would be better at doing that.’* (ID24;<5 years’ experience; group medical practice)

Consequently, most of the GPs had a degree of flexibility and choice in relation to their involvement in melanoma management, in terms of their usual clinical practice and care given to any one patient. This meant that for GPs who did choose to be actively engaged in melanoma management, there was then the need to decide not only when to refer a patient (if at all), but also to whom they would refer. For most GPs, referral options typically included another GP, a dermatologist, and a general or plastic surgeon. For those located in major metropolitan centres, referral could also be to surgical oncologists and multidisciplinary teams within melanoma units (Figure 2). Most of the GPs reported multiple referral options; however, those who worked regionally indicated that their location meant access to a particular specialist (for example, a dermatologist, plastic surgeon, or surgical oncologist) might be difficult or costly for their patients. A number of GPs from regional areas reported that should a patient indicate that they could not afford the time or money to see a specialist, they would agree to manage the patient themselves rather than refer.

#### Clinical practice (meso) level

At the practice level, GPs’ confidence and engagement in melanoma management appeared to be closely related to opportunities for informal, on-the-job training and attendance at training courses run by professional colleges and organisations or healthcare education providers. Reasons reported for not engaging in formal training included cost and availability of training programmes.

Considerable variation was reported in the amount of melanoma-related training and support GPs had received as registrars, which consequently affected the degree of comfort and confidence GPs had in managing patients with suspected or confirmed melanoma.

‘*I was pretty lucky to work in a practice with two long-term rural GPs that were pretty confident with their management of all types of skin cancers, so I got to learn a lot of skills from that, but I also had those GPs there to refer to for second opinion and management of complicated lesions* [and] *last year I had a lot of informal education using the dermatoscope from the GP supervisors*.’ (ID31;<5 years’ experience; independent GP)

Critically, a number of GPs (see Table S1) indicated that limited opportunities for training appeared to affect confidence in using a dermatoscope, which in turn appeared to influence their usual practice, with some GPs reporting that they did not use a dermatoscope at all; in contrast, others reported that they would use a dermatoscope for every skin assessment.

For some GPs, having a GP colleague within the practice with a special interest in melanoma (and more experience with a dermatoscope) meant there was always someone to whom they could quickly and easily refer patients for skin checks, biopsies and excisions. One consequence of this may be that for GPs with limited confidence in managing melanoma there is no imperative to develop knowledge or skills in melanoma management, allowing them instead to focus on other areas of general practice.

#### Individual practitioner and patient (micro) level

A small number of GPs (three of 23 interviewed) indicated that they essentially had little interest in management of patients with melanoma, that they did not feel comfortable with the ‘high stakes’ nature of melanoma management, and that they preferred to refer patients to a specialist rather than manage patients themselves. For some, their interest in melanoma management had been shaped early on by what they had been told about the difficulty of diagnosing melanoma:

*‘I kind of stopped doing skin cancer medicine all together because when I was a registrar, one of my colleagues who was a qualified GP at the time said to me, “you know, dermatologists have a 40 per cent risk of missing a melanoma” and I just thought, “geez, I don't want to be involved in this stuff.”‘* (ID55; 6–10 years’ experience, group medical practice)

**Figure 3.**
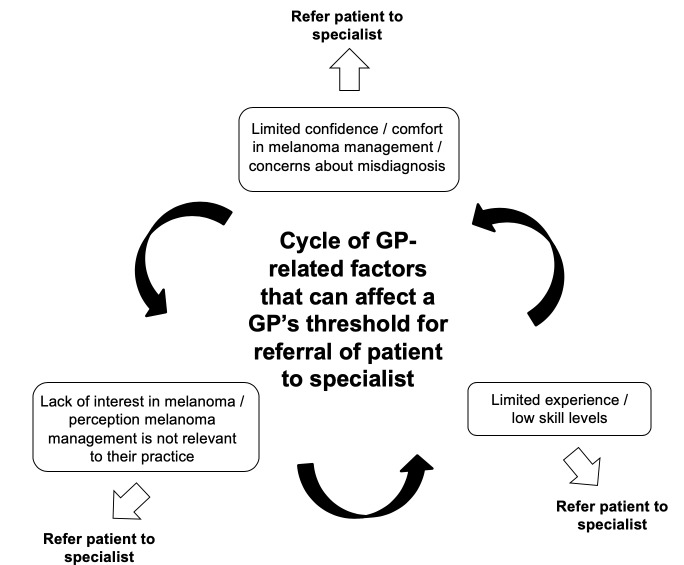
Factors that can affect a GP’s threshold for referral of a patient to a specialist

In contrast, others, while acknowledging that there would always be the unusual and atypical lesions that are challenging to pick up, were comfortable managing most patients who presented with a suspicious melanocytic lesion. As illustrated in Figure 3, it is possible that low levels of confidence in melanoma translated into less opportunity or desire to develop skills, knowledge, and experience in melanoma management, which, in turn, perpetuated concern about misdiagnosis, reinforcing the low levels of interest in melanoma. Experience in melanoma management was also affected by the patient population and practice location, with GPs commenting that valuable experience had been gained while working in rural practices where the patient population was *'* *older* *, whiter* *,* *and more sun exposed* *'* (ID31;<5 years’ experience; independent GP).

Many of the GPs indicated that clinical factors such as the location or size of a suspected or confirmed melanoma, and the interplay of these factors, would affect their decision to biopsy or excise, as would patient characteristics such as age and sex. A high index of suspicion that the lesion was a melanoma would also influence the decision to biopsy or refer immediately:

*‘*[As to whether or not I’d biopsy] *would depend on location, but probably yes, I would feel comfortable to do that. If it was in a location I didn't feel comfortable cosmetically or the lesion was very large or multiple lesions, I would tend to refer on, or if it looked really, really nasty.’* (ID30; 11–20 years’ experience, independent GP)

For the GP who had elected to perform the biopsy, receipt of the pathology report and confirmation that the lesion was a melanoma was often a clear trigger for referral, with many referring all melanomas regardless of whether they were in situ or invasive. In contrast, some of the GPs who saw higher volumes of patients (typically GPs working in skin cancer clinics but also several GPs in mainstream general practice) were comfortable performing the wide local excision on a confirmed melanoma. Another trigger for referral was the need for consideration of a sentinel lymph node biopsy

*‘If it's a thin melanoma, I would be confident to manage myself, but if they needed a node biopsy, if they needed really big excisions, then I would send them into the surgeons.’* (ID135; 6–10 years; government clinic)

Finally, the decision to refer was sometimes influenced by patient preferences. Some GPs referred patients who could afford to see a plastic surgeon for lesions, in the belief that they were likely to get a better outcome. In contrast, some regional GPs were also prepared to perform a wide local excision on patients they would normally refer on to a surgeon if cost was an issue for the patient, or if the patient was reluctant to travel to access public services.

## Discussion

### Summary

For the first time, this study provides a detailed account of the considerable variation in levels of confidence Australian GPs have in diagnosing and treating patients with suspected or confirmed melanoma, and of the variation that exists in Australian GPs’ preferred levels of involvement in melanoma management. This study also offers an explanation of why this variation exists, and identifies factors operating at the health system (macro), practice (meso), and GP and patient (micro) levels that contribute to this variation. The study indicates that low levels of confidence and experience in melanoma diagnosis and care can result in concerns about misdiagnosis, which, in turn, can further reduce interest, confidence, and experience in melanoma. Taken together, these data provide an explanation for why GPs may decide to refer or manage patients with suspected or confirmed melanoma.

### Strengths and limitations

A strength of this study is the breadth and richness of the data generated in the in-depth interviews. This was in part owing to the inclusion of GPs from a range of clinical settings (that is, GPs working in group and independent practices, as well as GPs working in skin cancer clinics), metropolitan and rural GPs, recently qualified GPs and those with many years of experience as a GP, those with an interest in skin cancer and those with a limited interest in skin cancer. A wide range of viewpoints were, therefore, explored. Limitations of the study include the possibility of selection bias toward GPs with an interest and expertise in skin cancer, given that some of the GPs were recruited at a skin cancer workshop. There was also a relatively small number of participants from regional and rural areas, so the range of experience of GPs working outside a metropolitan centre may not have been fully captured.

### Comparisons with existing literature

These findings extend what is already known about the complexity and diversity of the clinical pathways for melanoma in Australia^14,15,31^ and the overlapping roles GPs, dermatologists, and surgeons have in performing surgical procedures such as wide local excision for melanoma.^15,27^ The finding that opportunities for formal and informal training affected GPs’ confidence and decisions around referral broadens understanding of the relationship between training and the decision to refer,^15^ making explicit the interconnectedness of interest and concern about misdiagnosis with training and confidence. The findings also highlight how GPs’ involvement in cancer care can extend well beyond screening, prevention, and supportive care to include provision of definitive treatment, an area that other Australian studies have not acknowledged.^1,32^

It is clear from the current study that, in relation to melanoma management, GPs are not a homogeneous group. Some of the GPs working within mainstream general practice (those who could be described as having a special interest in skin cancer) had more in common with GPs working in skin cancer clinics than they did with generalist GPs. This was most noticeable in their attitudes towards melanoma management, decisions around managing in-house versus referring on, and their levels of confidence. The similarities identified in the current study reflect what has been reported about the practice, volume, and quality of skin cancer care provided by GPs who sub-specialise compared with generalist GPs.^33,34^ In particular, 2015 workforce data indicate that in Australia more than 50% of skin cancer services are provided by less than 4% of GPs who work within skin cancer clinics or who have a special interest in skin cancer.^33^ These sub-specialised GPs have also been reported to have a significantly lower melanoma numbers needed to treat (number of suspicious melanocytic lesions excised to detect one melanoma) than generalist GPs.^34^ Taking all of this together, the current study provides new data to support previous observations that *‘the role of such sub-specialised GPs should be defined and the factors associated with their higher performance, including training in dermatoscope use, should be promoted* *’*.^34^ A greater understanding of the factors associated with higher performance would be useful both to GPs who wish to become more involved in melanoma management and to GPs with a special interest in skin cancer or already employed within skin cancer clinics.

### Implications for research and practice

GP involvement in melanoma patient care can extend well beyond the widely recognised roles of cancer screening, prevention and supportive care to include provision of definitive melanoma patient management. GPs with an interest in being involved in melanoma patient management should be encouraged and supported to develop the skills needed to manage patients with suspected or confirmed melanomas; importantly, they should also be given information that will allow them to decide when referral to a specialist is appropriate.
